# Simulation methods for multiperiodic and aperiodic nanostructured dielectric waveguides

**DOI:** 10.1007/s11082-017-0918-6

**Published:** 2017-02-16

**Authors:** Moritz Paulsen, Lars Thorben Neustock, Sabrina Jahns, Jost Adam, Martina Gerken

**Affiliations:** 1 Institute of Electrical Engineering and Information Technology, Kiel University, Kaiserstr. 2, 24143 Kiel, Germany; 2 Electrical Engineering, Stanford University, 350 Serra Mall, Stanford, CA 94305-9505 USA; 3 Mads Clausen Institute, University of Southern Denmark, Alsion 2, 6400 Sønderborg, Denmark

**Keywords:** Deterministic aperiodic nanostructures, Waveguide gratings, Waveoptic simulations

## Abstract

Nanostructured dielectric waveguides are of high interest for biosensing applications, light emitting devices as well as solar cells. Multiperiodic and aperiodic nanostructures allow for custom-designed spectral properties as well as near-field characteristics with localized modes. Here, a comparison of experimental results and simulation results obtained with three different simulation methods is presented. We fabricated and characterized multiperiodic nanostructured dielectric waveguides with two and three compound periods as well as deterministic aperiodic nanostructured waveguides based on Rudin–Shapiro, Fibonacci, and Thue–Morse binary sequences. The near-field and far-field properties are computed employing the finite-element method (FEM), the finite-difference time-domain (FDTD) method as well as a rigorous coupled wave algorithm (RCWA). The results show that all three methods are suitable for the simulation of the above mentioned structures. Only small computational differences are obtained in the near fields and transmission characteristics. For the compound multiperiodic structures the simulations correctly predict the general shape of the experimental transmission spectra with number and magnitude of transmission dips. For the aperiodic nanostructures the agreement between simulations and measurements decreases, which we attribute to imperfect fabrication at smaller feature sizes.

## Introduction

**Fig. 1:**
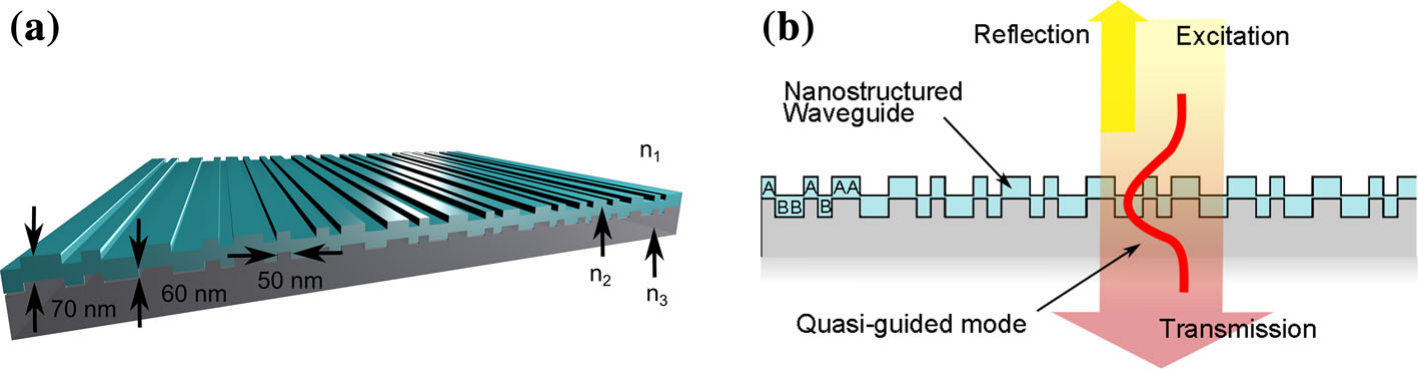
Simulation model for nanostructured dielectric waveguide, here with a Thue-Morse nanostructure. **a** 70 nm high-index, waveguiding TiO_2_ (n_2_) layer on top of 60 nm deep structure in AMONIL (n_3_ = 1.52) substrate. **b** Excitation of quasi-guided mode (QGM) with normal-incidence illumination, As and Bs showing the translation of the binary sequence into a nanostructure

Grating waveguides have been studied throughout the last decades and show high potential for integrated sensing applications (Rosenblatt et al. 1997; Threm et al. 2012). They have been studied for label-free biosensing (Cunningham et al. 2015; Jahns et al. 2015; Nazirizadeh et al. 2016), to increase the light outcoupling from organic light-emitting diodes (Kluge et al. 2014) and to improve the efficiency of solar cells (Zeng et al. 2006). Grating waveguides are planar waveguides with embedded diffractive grating structures. Figure 1 depicts a TiO_2_ waveguide with aperiodic deterministic nanostructure based on a Thue–Morse binary sequence. Deterministic aperiodic nanostructures are engineered ordered nanostructures without periodicity (Dal Negro 2012a, b; Maciá 2012). Compound multiperiodic gratings and deterministic aperiodic nanostructures offer the opportunity to tailor the spectral properties and have recently been suggested for refractive index biosensing (Boriskina et al. 2008a, b; Kluge et al. 2014; Neustock et al. 2016). The grating structure allows incident light to couple to guided modes by scattering. The guided light can again couple out due to the nanostructure and thus the modes are called quasi-guided modes (QGM) or leaky modes. We investigate the case that normally incident light is coupled into and out of QGM in the waveguide structure as depicted in Fig. 1b. Reemission of the QGM in the reflection direction leads to characteristic guided-mode resonances (GMR) in the transmission spectrum, which depend on the angle of incidence, polarization of the light, refractive index of the material and the geometric properties, such as the nanostructure sequence, duty cycle and structure depth (Fan and Joannopoulos 2002). In this work we employ and compare three different simulation methods—finite element method (FEM, COMSOL Multiphysics^®^ Wave Optics Module by COMSOL Inc.), finite difference time domain (FDTD, FDTD Solutions by Lumerical Solutions, Inc.) and rigorous coupled wave analysis (RCWA, in-house implementation)—for simulating the transmission properties of compound multiperiodic and deterministic aperiodic nanostructures. Five different structures are examined—two multiperiodic structures (one with a two-compound and one with a three-compound grating) and three binary deterministic aperiodic sequences with different degrees of disorder (Thue–Morse, Fibonacci and Rudin–Shapiro).

The binary deterministic aperiodic sequences are obtained by simple mathematical substitution rules and offer different degrees of disorder in their spatial Fourier spectrum (Dal Negro and Boriskina 2012a). Compound gratings are obtained by a superposition of multiple monoperiodic gratings. A logical disjunction operation is performed. Each further superimposed grating adds peaks corresponding to its period to the spatial Fourier spectrum. Thus, compound multiperiodic gratings allow the design of GMRs with arbitrary wavelengths in the transmission and reflection spectrum. By tuning the duty cycle also the relative intensities of the GMRs may be tailored (Kluge et al. 2014). Dielectric waveguides with compound multiperiodic and deterministic aperiodic nanostructures offer a plentitude of degrees of freedom in the design of transmission and reflection spectra for applications in the above mentioned areas. For example, the design of integrated sensor chips with more redundancy by using multiple resonances will help to create more reliable systems. The ability to simulate and design nanostructures for specific applications is crucial. To help future designers choose their simulation tools, we here present a comparative study of three simulation methods and compare the simulation results to experimental results. This work is structured as follows. We first introduce the structures under investigation in Sect. 2. Section 3 describes the simulation methods. Also the fabrication process of the samples and the measurement setup are detailed in this section. In Sect. 4, the simulation and measurement results are shown. The three simulation methods are compared to each other and to the measured data.

## Structures under investigation

Five different structures are under investigation in this work—two multiperiodic and three aperiodic structures. The details of the different structures are shown in Table 1. The multiperiodic structures are two basic examples, one disjunction of two and one of three different monoperiodic gratings, following the approach of Kluge et al. (2014). The periodicities of 250, 300, and 350 nm were chosen to show distinctive resonances in the visible wavelength range, being exemplary for any disjunction of different periodicities. We expect the two multiperiodic structures to show two and three main resonance features in the transmission spectrum at normal incidence as follows from Bragg theory (Rosenblatt et al. 1997):

1 $$ \lambda_{res,i} = \Lambda_{i} \times \left( {n_{eff} \pm { \sin }\left( \Theta \right)} \right) $$

here $\lambda_{res,i}$ is the wavelength of the quasi guided mode resonance of the ${\text{i}}_{\text{th}}$ grating component, with the period $\Lambda_{i}$. $n_{eff}$ is the effective refractive index of the guided mode at resonance and $\Theta$ is the angle of incidence. For normal incidence, the spectral resonance position is a function of the grating component $\Lambda_{i}$ and the effective refractive index $n_{eff}$, which depends on the material’s refractive index and the geometry of the high-index layer. All incorporated materials are modeled as non-magnetic, lossless (no complex refractive index) materials, and the top and bottom layers are assumed to be non-dispersive, with constant refractive indices n = 1 and n = 1.52 for air and AMONIL, respectively.

**Table 1:** Overview of simulated and fabricated nanostructures

Name	Type	Description	Supercell length, number of recursions
2-compound	Compound multiperiodic, two periods	Λ_1_ = 250 nm, Λ_2_ = 300 nm duty cycles, t_1_ = 0.3, t_2_ = 0.4	L = 1500 nm
3-compound	Compound multiperiodic, three periods	Λ_1_ = 250, Λ_2_ = 300 nm, Λ_3_ = 350 nm, duty cycles: t_1_ = 0.3, t_2_ = 0.3, t_3_ = 0.3	L = 10,500 nm
Rudin–Shapiro	Deterministic aperiodic, continuous spectrum	Substitution: AA → AAAB, AB → AABA, BA → BBAB, BB → BBBA	L = 12,800 nm, N = 7
Thue–Morse	Deterministic aperiodic, singular continuous spectrum	Substitution: A → AB, B → BA	L = 12,800 nm, N = 9
Fibonacci	Deterministic aperiodic, pure-point spectrum	Substitution: A → AB, B → A	L = 11,600 nm, N = 13

The aperiodic structures are binary sequences following simple mathematical substitution rules. The three sequences are based on the Rudin–Shapiro, Thue–Morse and Fibonacci substitution rules, which are chosen for their different degrees of disorder, ranging from a pure-point spatial Fourier-spectrum (Fibonacci) to a continuous spatial Fourier-spectrum (Rudin–Shapiro) (Dal Negro and Boriskina 2012a). The length of the supercell, which we define as the geometric structure, which includes the binary sequence once in the aperiodic case and has the length of the least common multiple of all the periods in the multi-periodic case. It depends on the number of recursions (N) of the respective substitution rule.

**Fig. 2:**
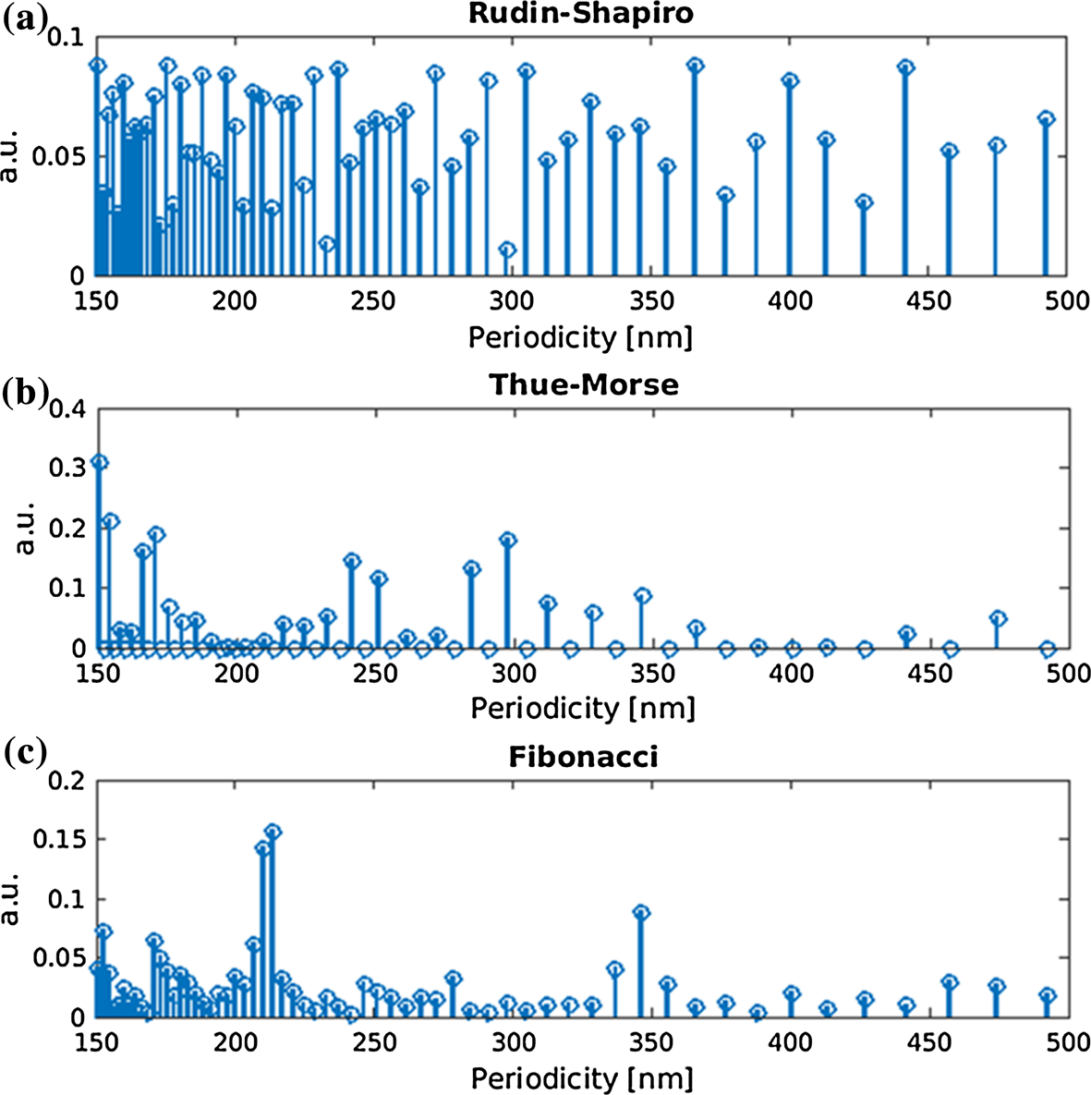
Fourier spatial spectra of deterministic aperiodic nanostructures with 50 nm feature width, **a** Rudin–Shapiro continuous spectrum, **b** Thue–Morse singular continuous spectrum, **c** Fibonacci pure-point spectrum

The aperiodic sequences are translated into nanostructures by substitution of each letter by either a ridge or a groove of 50 nm in the substrate layer (see Fig. 1b). Figure 2 shows the resulting calculated Fourier coefficients for spatial periodicities ranging from 150 to 500 nm. The Fourier coefficients may be interpreted as likeliness to monoperiodic gratings. Following Eq. 1, peaks in the Fourier spectrum will add GMRs to the transmission and reflection spectrum. The chosen range of periodicities in Fig. 2 will cover all resonance wavelengths in the visible spectrum. The Rudin–Shapiro nanostructure has a flat, almost continuous spectrum with regard to the finite length of the calculated sequence. The Thue–Morse nanostructure has some clusters of higher Fourier components at 170, 250, and around 300 nm periodicities, being singular continuous. Finally, the Fibonacci nanostructure shows a single point spectrum having its peaks around 215 and 345 nm.

**Fig. 3:**
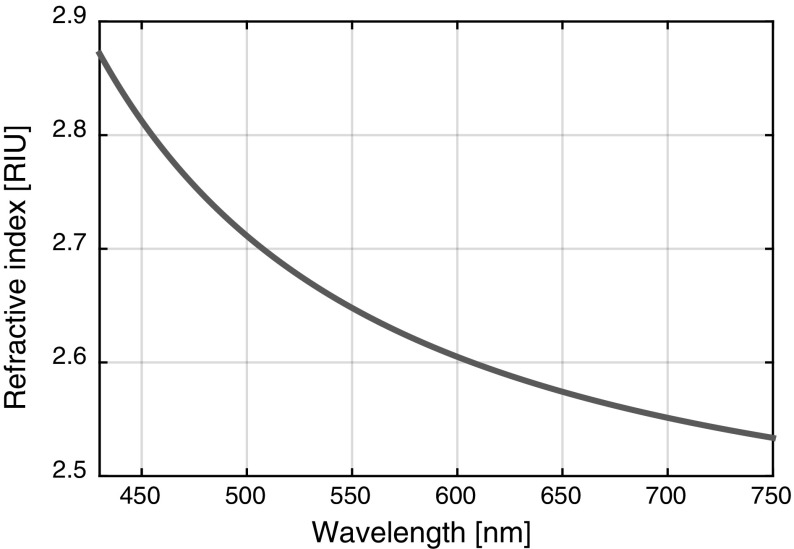
Refractive index dispersion of titanium dioxide [Devore51]

The implemented models were designed to embody the fabricated samples, which consist of a nanostructured substrate on which a high-index layer is sputtered. The implemented structure has a depth of 60 nm in the substrate and a 70 nm high-index layer is added on top. The characteristic dispersion relation of TiO_2_ is used as depicted in Fig. 3 (Devore 1951). The cladding refractive index is set to air (n_1_ = 1) and the substrate is assumed to be AMONIL photo resist (n_3_ = 1.52).

## Methods

We employ three different simulation methods for the extraction of (frequency-domain) reflection and transmission, as well as near-field data: Finite element method (FEM), rigorous coupled wave analysis (RCWA) and finite difference time domain (FDTD) method. All three methods solve the Maxwell’s equations, with lateral periodic boundaries and vertical radiating boundaries. In the case of non-magnetic materials this is equivalent to the solution of Helmholtz’ (wave) equation. While the former two methods operate completely in the frequency domain, FDTD is a time-domain technique. In time domain, in the case of time-harmonics electromagnetic fields, Maxwell’s equations are again equivalent to the wave equation, with respect to the well-known phasor definition (see (Jackson 1998; Taflove and Hagness 2005) for details). In consequence, all three methods tackle equivalent sets of partial differential equations, while in FDTD the desired frequency data can be efficiently extracted via fast Fourier transform (FFT). All three methods implement the structures as periodic super-cells with lateral Bloch-periodic boundary conditions. For the calculation of the transmission coefficients, all orders of diffraction are integrated.

The simulation regime is set from 430 to 750 nm with 0.5 nm resolution. This range covers the part of the visible spectrum, in which the broadband illumination source of the measurement setup has sufficient power to take reliable spectra. With the structures being invariant in the in-plane direction of the grating structures, two-dimensional simulations are carried out. The excitation polarization is chosen for the transverse-electric (TE) case, having the electric field vector in the plane of the waveguide.

In the following, the method-specific implementations alongside sample fabrication and the measurement setup are described in further detail.

### FDTD

The FDTD model consists of a two-dimensional (2D) unit cell of supercell width, that comprises the different grating types implemented as polygons. The gratings are sandwiched in vertical direction by a cladding layer (870 nm) on top and the substrate layer (400 nm) at the bottom. To implement the necessary radiating boundary conditions, the cell is sandwiched by 48-layer perfectly matched layers (PML). Horizontally the cell is sandwiched by periodic (Bloch) boundary conditions, mimicking an infinite repetition of the supercell. This structure is excited by a normally incident plane wave with a sine-modulated Gaussian intensity profile. This excitation allows for the extraction of broadband frequency information from a single time-domain solver run. For a controllable accuracy, the grating region (±200 nm in vertical direction) is discretized with square Yee-Cells with a fixed side length. Outside this region, to increase computation speed, we allow a (vertical) widening of the mesh cells towards the boundaries. A convergence analysis showed that a unit cell size of 2.5 nm is sufficiently small to guarantee accurate results. After running the simulation, the desired frequency-domain information can be obtained by a Fourier transformation with respect to the desired frequency discretization and bandwidth. In the present showcase, we performed FDTD runs with an impinging transverse electric (TE) plane wave, comprising a wavelength (free-space) range from 430 to 750 nm, discretized in 0.5 nm steps. The transmission/reflection data are collected by a horizontal line field monitor, spanning the entire simulation cell width, located between the plane wave source and the PML, while the local field data are collected via two-dimensional field monitors surrounding the grating region.

### RCWA

The in-house RCWA implementation is implemented in Matlab^®^ and based on the algorithms presented in Moharam et al. (1995a, b), suitable for an RCWA implementation for a geometry with different layers. To model the geometries shown in Fig. 1, they are divided in three layers. An upper layer modelling the variation between high index and cladding material, a middle layer consisting only of high-index material and a lower layer describing a variation of refractive index between high-index and substrate material. For each layer, the corresponding Fourier coefficients of the multiperiodic and aperiodic gratings are calculated analytically with a base frequency of the inverse length of the supercell. The number of Fourier coefficients for each grating is determined by a convergence analysis. For the aperiodic gratings and the multiperiodic grating with 3 superimposed periods, 512 Fourier coefficients are sufficient, whilst for the multiperiodic grating consisting of 2 periods only 64 Fourier coefficients are necessary. Generally, the number of required coefficients increases with smaller feature sizes with respect to supercell length. After implementing the geometry, the spectrum is calculated for each wavelength individually.

To generate the near-field plot, the solution of the linear system underlying the RCWA is used to reconstruct the field as a superposition of plane waves, according to the equations given in (Moharam et al. 1995b) for the derivation of the algorithm.

### FEM model

**Fig. 4:**
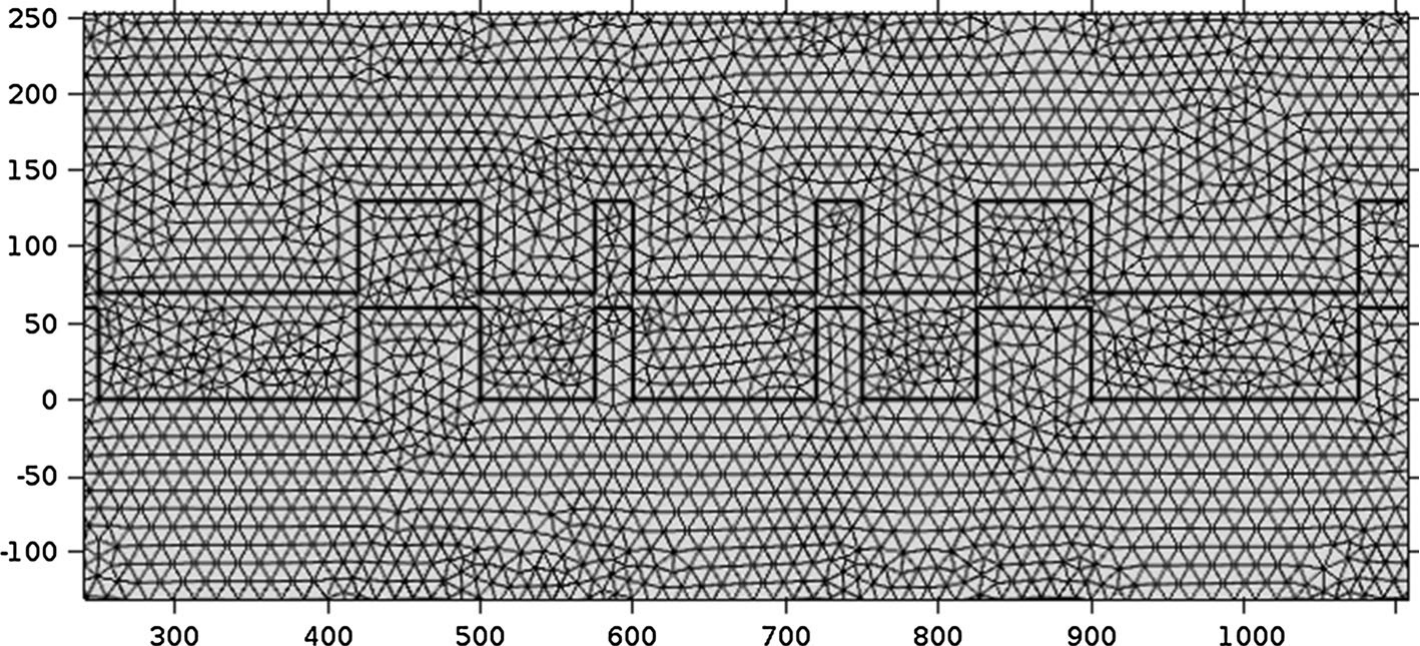
Triangular mesh of the COMSOL finite element simulation, showing the two-compound multiperiodic grating structure

The COMSOL model features a polygon high-index block with wavelength dependent refractive index as specified above. The cladding and substrate layers are 700 nm thick, with additional 300 nm perfectly matched layers (PML) which are backed by second order scattering layers. A mode excitation is introduced by a port at the upper boundary to the PML. The transverse electric wave has an amplitude of 1 V/m and travels towards the nanostructure with its wave vector orthogonal to the plane of the waveguide layer (normal incident). A triangular, non-uniform mesh is user specified with minimum and maximum element sizes of 2.5 and 15 nm, which showed good convergence for both transmission and reflection spectra. A section of the mesh is shown in Fig. 4. The normalized power flow is integrated 600 nm above and below the high index layer for calculation of transmission and reflection properties. The simulation is solved for the out of plane, transverse electric (TE) field, at incident wavelengths of 430–750 nm as specified above. The electrical field is exported to Matlab and interpolated on a 1 nm rectangular grid via the *mphinterp()* method.

### Fabrication and measurement setup

To fabricate the compound multiperiodic and aperiodic nanostructured dielectric waveguides, first glass substrates are cleaned with acetone and isopropanol in an ultra-sonic bath for 15 min each and are dehydrated at 160 °C for 10 min. 150 µl adhesion promoter (Amoprime, *AMO GmbH*) is spincoated onto the substrate at 3000 rpm for 30 s. After baking the substrates at 110 °C and cooling them for 2 min each, 150 µl photoresist (AMONIL, *Amo GmbH*) is spincoated at 3000 rpm for 30 s. A thin film of about 200 nm is formed this way, into which the nanostructure is transferred by a nanoimprint process. AMONIL has the same refractive index as the glass substrate to prevent interference effects during illumination. A polydimethylsiloxane (PDMS) stamp, which is a replica of a nickel shim containing the original electron-beam-written nanostructures (60 nm structure depth, *Karlsruhe Nano and Micro Facility*), is carefully pressed to the photoresist to transfer the structure. For fabrication details of the PDMS stamp, see (Jahns et al. 2015). The photoresist is UV hardened through the PDMS stamp for 80 s. After removing the stamp, a 70 nm thick titanium dioxide high-index layer is deposited by reactive sputtering, to form the waveguiding layer.

The fabricated samples are placed into a transmission confocal microscope setup. A polarization filter is used to excite either transverse electric (TE) or transverse magnetic (TM) resonances. The transmitted light is coupled to a spectroscope (Shamrock 500i, Andor) with a CCD camera (Andor). A halogen broadband illumination source is used. To obtain the transmission coefficients of the samples, the measured spectral data is normalized to the system response of the setup.

## Results

In this section the simulation results as well as the measured transmission spectra are presented. The three methods are compared in different scenarios.

### Near field simulations

**Fig. 5:**
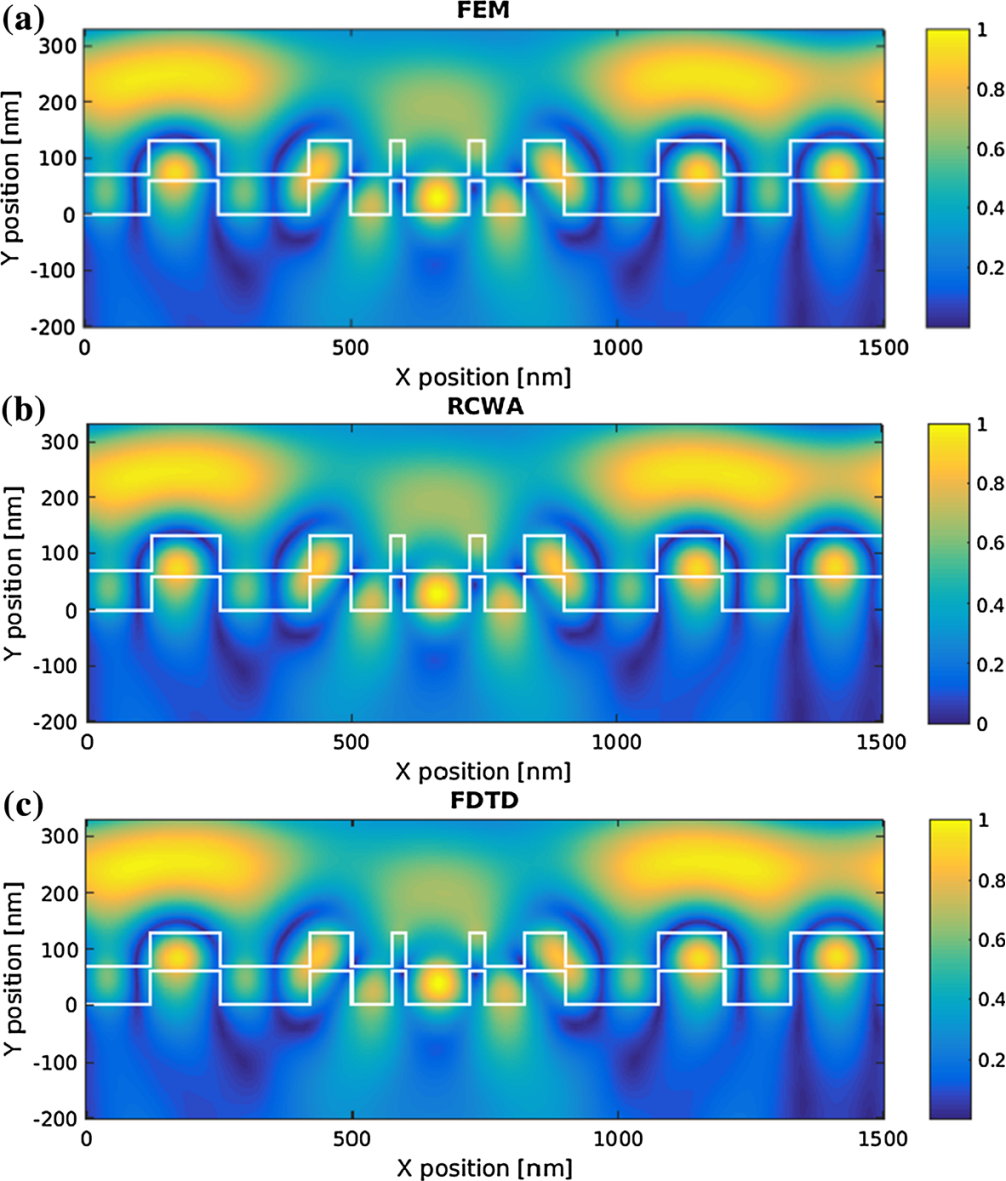
Computed normalized electric field strengths for the 2-compound grating structure. **a** FEM, **b** RCWA, **c** FDTD

**Fig. 6:**
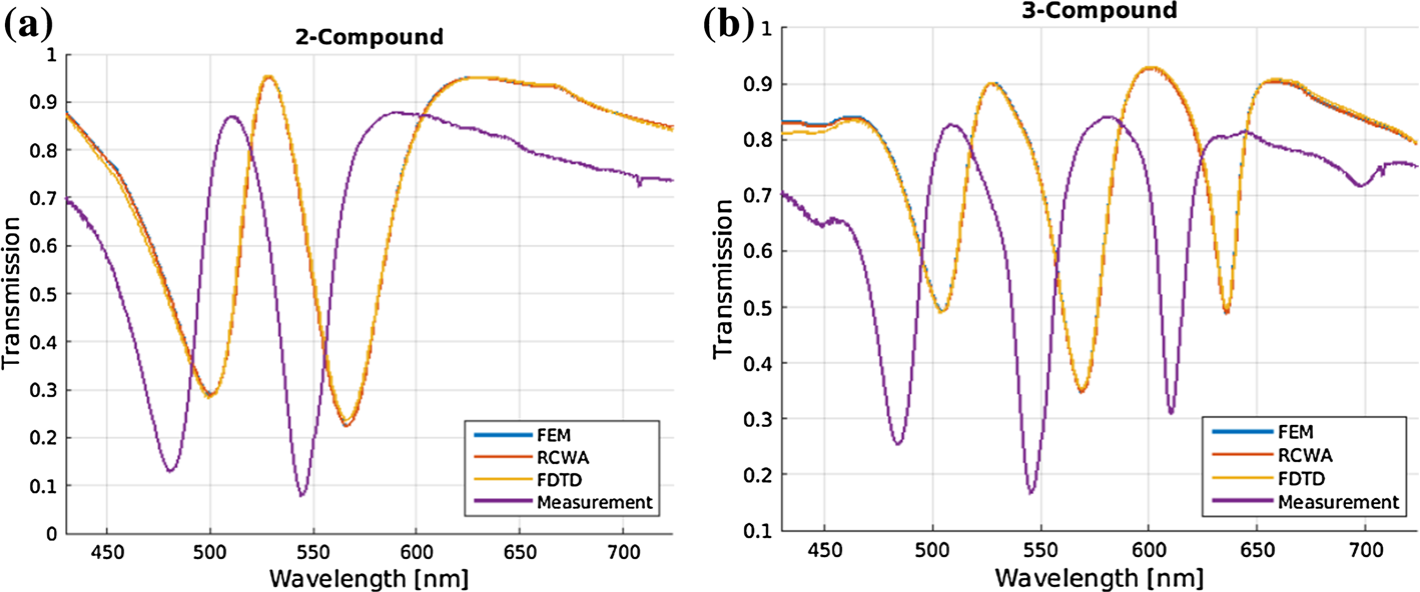
Simulated and measured transmission spectra of dielectric waveguides with aperiodic nanostructure based on **a** 2-compound and **b** 3-compound multiperiodic nanostructures. Three different simulation methods are employed

All three simulation methods are used to calculate near fields of the structures. The 2-compound structure is chosen to be shown here due its shorter supercell length of 1500 nm. The magnitude of the electrical field strength is calculated at the first spectral resonance (498.5 nm, see Fig. 6) for the length of a single supercell. The calculated near fields are depicted in Fig. 5. Outlines of the high refractive index layer are plotted in white, to show the geometrical structure.

All three methods are capable of calculating the electric field of both multiperiodic and aperiodic structures. The calculated fields only show small differences. The field distribution shows the resonant behavior of the 498.5 nm wavelength with regions of concentrated field energy. Due to the distinctive meshing of the methods, disparity plots mainly reveal interpolation errors.

### Multiperiodic nanostructures

For all five structures, transmission spectra are computed and are compared in the following. First the results of the two multiperiodic structures are presented. Figure 6 shows the simulated transmission spectra for the 2-compound (left) and 3-compound (right) nanostructured waveguides. The three simulation methods have very similar results, showing two major dips at 498.5 and 560 nm. Both the simulated 2-compound and 3-compound transmission spectra predict the general form of the measured data well. The simulated results are redshifted by about 15 nm compared to the measurement. This mismatch might be attributed to an imperfect TiO_2_ layer, having a lower refractive index than bulk TiO_2_ as found in the literature (Devore 1951). No experimental dispersion relation of the sputtered TiO_2_ is available at the moment. Another reason for the spectral mismatch might be a lower height of the high index layer as a result of the sputtering process, which showed to have an accuracy of a few nanometers. The transmission of the measurement is lower in general, compared to the simulation, which we attribute to material absorption and additional scattering at the imperfect material boundaries. Even though the measurement and simulation are not perfectly matched, the results show, that the general behavior—the resonance position and shape—of multi-periodic nanostructures can be predicted by all three methods. Knowledge of the exact material parameters seems to be crucial in absolute prediction of the spectral position and transmission values.

### Aperiodic nanostructures

**Fig. 7:**
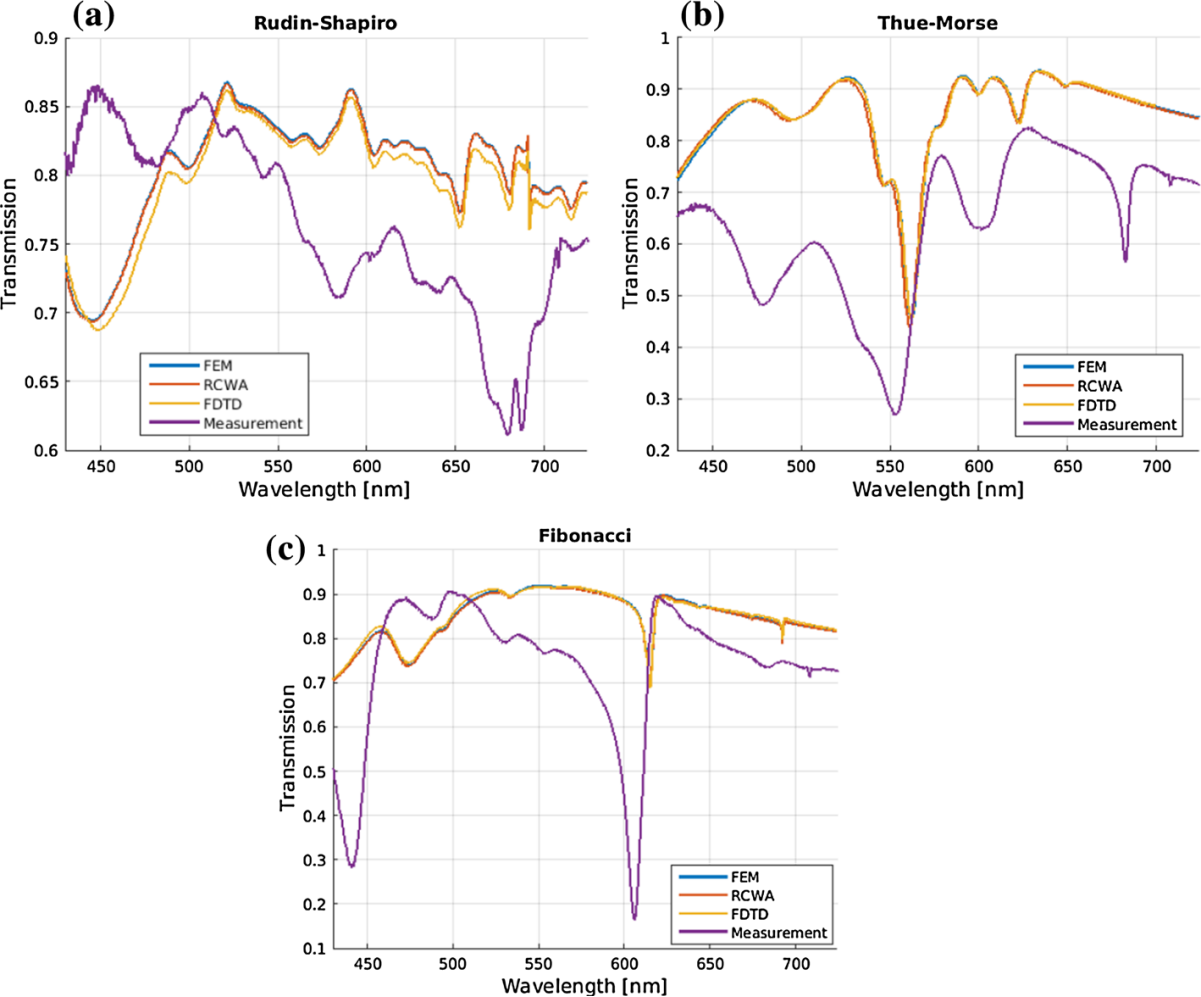
Simulated and measured transmission spectra of dielectric waveguides with aperiodic nanostructure based on **a** Rudin–Shapiro, **b** Thue–Morse, and **c** Fibonacci sequence. Three different simulation methods are employed

Figure 7 shows the computed transmission spectra for the Rudin–Shapiro, Thue–Morse, and the Fibonacci aperiodic nanostructures as specified above. Here, the three simulation methods are again in good agreement. Only in the case of the Rudin–Shapiro nanostructure the spectrum of the FDTD simulation shows lower transmission.

The general features of the simulated transmission spectra are contained in the measured data. The resonance dips of the measurement are much broader than the simulated. This effect might be caused by the small feature size (50 nm) of the ridges and grooves of the nanostructure, which is at the lower limit of the current fabrication possibilities. Thus, the imperfect fabrication lowers the quality of the resonances. Again the transmission spectrum of the measurement is shifted to the blue regime. In the case of the Rudin–Shapiro nanostructure, due to the high number of resonances and the imperfection of the fabrication, an accurate prediction of the transmission spectrum was not possible. Due to the lack of short range correlations of this deterministic structure with continuous Fourier spectrum, the analysis of the Rudin–Shapiro sequence has been reported complicated (Dal Negro and Boriskina 2012a).

### Comparison of simulation methods

While all the methods are suitable for the simulation of transmission spectra and near fields for light-matter interaction in multiperiodic and aperiodic nanostructured dielectric waveguides, each method has its own benefits and drawbacks.

For the comparison of the methods, the computational times for three scenarios are computed on the same computer (dual Intel^®^ Xeon^®^ CPU E5-2637 v3 @ 3.50 GHz, 512 GB of RAM). The first scenario is the computation of the transmission spectrum over the visible range as shown above (430–750 nm, 641 spectral points). The second scenario is the electrical field calculation as shown in Fig. 5 for a single wavelength and the 2-compound structure detailed in Table 1. The third scenario is the computation of both transmission and near-field data for the entire spectrum. The FDTD simulations and the FEM simulations are natively parallelized. We have parallelized the in-house RCWA code with Matlab^®^’s parallel processing toolbox. For the present comparison, we restricted all concurrent simulations to 8 workers (CPU cores).

From the simulation times in Table 2 it follows that the RCWA method is best suited for the calculation of transmission and reflection spectra, as these data are not deduced from local fields (as it is the case in FEM-based calculations). On the other hand, FEM turns out to be efficient in calculating single field images, and COMSOL Multiphysics^®^ offers a broad range of tools for subsequent analysis. The broadband nature of FDTD leads to the shortest simulation time if one is interested in full spectra alongside field plots for all involved wavelengths. On the other hand, this nature also leads to significant time drawbacks, when exercising the first two scenarios, as compared to the frequency domain methods. For extensive parameter sweeps, in RCWA, less Fourier components may be used to have an even faster calculation with less details. Concurrently, an approach with lower spectral and meshing resolution in the COMSOL^®^’s Wave Optics Module and FDTD Solutions is thinkable to reach shorter simulation times. The 2-compound structure under investigation is the smallest structure we investigated in this study. Simulation time will scale with the number of Fourier coefficients in the RCWA case and with the size of the supercell (number of mesh elements) in the FEM and FDTD case. Please note that our in-house implementation has still potential for further runtime optimization (Hench and Strakoš 2008).

**Table 2:** Comparison of simulation methods for multiperiodic and aperiodic nanostructured dielectric waveguides

Method	Scenario 1: transmission spectrum	Scenario 2: single- wavelength field	Scenario 3: full Spectrum and field	Pros	Cons
Finite Element Method (FEM, COMSOL Multiphysics^®^ Wave Optics Module)	Slowest, all fields have to be calculated, 13 min, 11 s	Fastest, 6 s	13 min, 11 s	Fast single field profile calculation; extensive toolbox	Proprietary
Finite Difference Time Domain (FDTD, FDTD Solutions by Lumerical Solutions, Inc.)	1 min 7 s	59 s	Fastest, 5 min, 4 s	Fast full-spectrum full-field calculation	Proprietary
Rigorous Coupled Wave Analysis (RCWA, in-house implementation)	Fastest, 18 s	12 s	7 min, 10 s	Fast spectrum calculation	Non-intuitive implementation, no user interface

## Conclusion

In conclusion we computed the transmission coefficients and near field characteristics of compound multiperiodic and deterministic aperiodic nanostructured dielectric waveguides with three different simulation methods and compared these to measured data. The three simulation methods, namely FDTD, FEM and RCWA give close results in both transmission spectra and near fields. In the multiperiodic cases a good prediction of the experimental transmission spectra is possible. Here, the spectral features are in good agreement with the measurements. For the aperiodic cases the predictive quality of the simulations was lower. In the case of the Thue–Morse and Fibonacci nanostructures the same general features were observed in both simulation and measurement. For the highly disordered Rudin–Shapiro sequence no prediction was possible. Each simulation method has its advantages and disadvantages. While all result in similar simulation results, the proprietary FEM and FDTD software packages allow for a comfortable implementation of different structures with little algorithmic knowledge. The in-house RCWA implementation on the other hand offers much faster simulation times.

## Figures and Tables

**Fig. 8:**
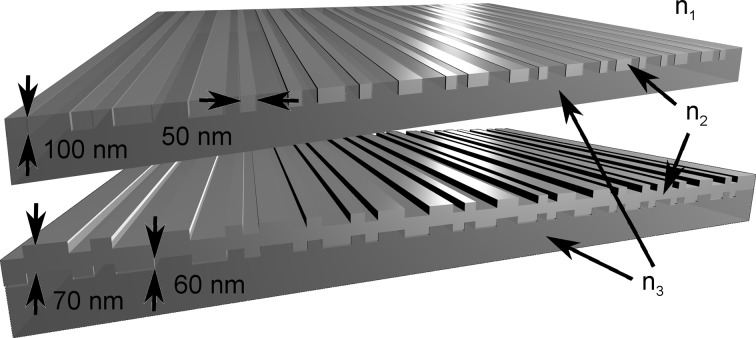
*Top* Simplified model with 100 nm embedded high-index ridges (n_2_ = 2.44) in AMONIL substrate. *Bottom* Model as introduced in Fig. 1

**Fig. 9:**
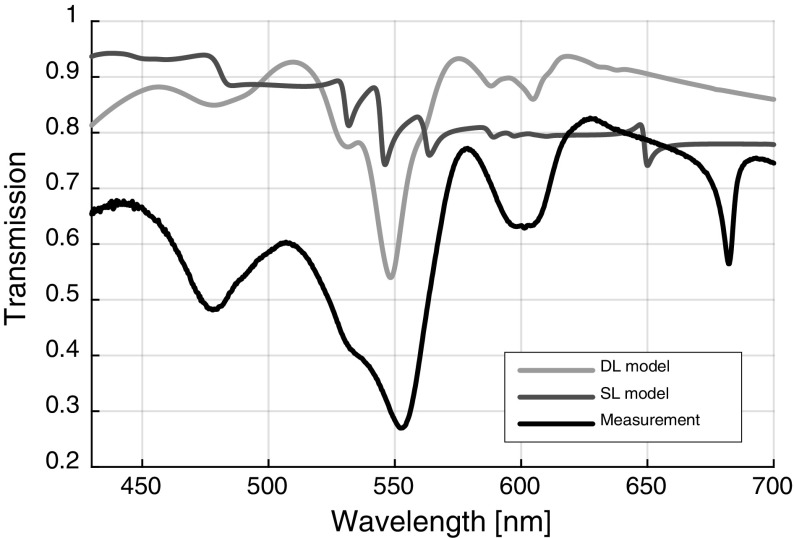
Comparison of FDTD simulation models single-layer (SL) and double-layer (DL) and measurement showing significant differences

## Appendix: comparison of different models

In a preliminary investigation, we tried a simplified model of the experimental structure for easier implementation. Here, we present the FDTD results comparing the behavior of the two different simulation models depicted in Fig. 8. Model 1 (single layer, SL) implements the waveguide layer as a single layer of corrugated refractive index. Model 2 (double layer, DL) on the bottom of Fig. 8 resembles the experimental structures more closely. The first single-layer (SL) approach models the high-index layer as 100 nm high-index (n_2_) ridges embedded in the substrate.

Both SL and DL simulations in this preliminary investigation have non-dispersive material properties with fixed refractive indices of n_1_ = 1, n_2_ = 2.44, n_3_ = 1.52. Figure 9 shows FDTD simulations of the transmission characteristics of the two models as well as the measured transmission of the Thue–Morse nanostructured waveguide. The refractive index of the high-index layer differs from the values used for Fig. 7, which includes material dispersion. This accounts for the differences in resonance position and shape with regard to Fig. 9.

The double layer simulations match better the measured data, showing the general spectral features of the measurement. As a result, the simplification introduced by the single layer model is not suited for the simulation of the investigated structures.

## References

[CR1] Boriskina SV, Gopinath A, Dal Negro L (2008). Optical gap formation and localization properties of optical modes in deterministic aperiodic photonic structures. Opt. Express.

[CR2] Boriskina SV, Dal Negro L (2008). Sensitive label-free biosensing using critical modes in aperiodic photonic structures. Opt. Express.

[CR3] Cunningham B, Zhang M, Zhuo Y, Kwon L, Race C (2015). Review of recent advances in biosensing with photonic crystal surfaces. IEEE Sens. J..

[CR4] Dal Negro L, Boriskina SV (2012). Deterministic aperiodic nanostructures for photonics and plasmonics applications. Laser Photonics Rev..

[CR5] Dal Negro L (2012). Enhancing optical biosensing with aperiodicity. SPIE Newsroom.

[CR6] Devore JR (1951). Refractive indices of rutile and sphalerite. J. Opt. Soc. Am..

[CR7] Fan S, Joannopoulos JD (2002). Analysis of guided resonances in photonic crystal slabs. Phys. Rev. B.

[CR8] Hench JJ, Strakoš Z (2008). The RCWA method—a case study with open questions and perspectives of algebraic computations. ETNA Electron. Trans. Numer. Anal. [electronic only].

[CR9] Jackson JD (1998). Classical electrodynamics.

[CR10] Jahns S, Bräu M, Meyer BO, Karrock T, Gutekunst SB, Blohm L, Selhuber-Unkel C, Buhmann R, Nazirizadeh Y, Gerken M (2015). Handheld imaging photonic crystal biosensor for multiplexed, label-free protein detection. Biomed. Optics Express.

[CR11] Kluge C, Adam J, Barié N, Jakobs PJ, Guttmann M, Gerken M (2014). Multiperiodic nanostructures for photon control. Opt. Express.

[CR12] Maciá E (2012). Exploiting aperiodic designs in nanophotonic devices. Rep. Prog. Phys..

[CR13] Moharam MG, Grann EB, Pommet DA, Gaylord TK (1995). Formulation for stable and efficient implementation of the rigorous coupled-wave analysis of binary gratings. J. Opt. Soc. Am. A:.

[CR14] Moharam MG, Pommet DA, Grann EB, Gaylord TK (1995). Stable implementation of the rigorous coupled-wave analysis for surface-relief gratings: enhanced transmittance matrix approach. J. Opt. Soc. Am. A:.

[CR15] Nazirizadeh Y, Behrends V, Prósz A, Orgovan N, Horvath R, Ferrie AM, Fang Y, Selhuber-Unkel C, Gerken M (2016). Intensity interrogation near cutoff resonance for label-free cellular profiling. Sci. Rep..

[CR16] Neustock, L.T., Jahns, S., Adam, J., Gerken, M.: Optical waveguides with compound multiperiodic grating nanostructures for refractive index sensing. J. Sens. 6174527 (2016). doi:10.1155/2016/6174527

[CR17] Rosenblatt D, Sharon A, Friesem AA (1997). Resonant grating waveguide structures. IEEE J. Quantum Electron..

[CR18] Taflove A, Hagness SC (2005). Computational electrodynamics: the finite-difference time-domain method.

[CR19] Threm D, Nazirizadeh Y, Gerken M (2012). Photonic crystal biosensors towards on-chip integration. J. Biophotonics.

[CR20] Zeng L, Yi Y, Hong C, Liu J, Feng N, Duan X, Kimerling LC, Alamariu BA (2006). Efficiency enhancement in Si solar cells by textured photonic crystal back reflector. Appl. Phys. Lett..

